# Fatty Acid Composition of a Maternal Diet and Erythrocyte Phospholipid Status in Latvian Pregnant Women

**DOI:** 10.3390/medicina59091514

**Published:** 2023-08-23

**Authors:** Ksenija Nikolajeva, Olga Aizbalte, Anna Piskurjova, Roberta Rezgale, Vinita Cauce, Dzintars Začs, Laila Meija

**Affiliations:** 1 Doctoral Department, Faculty of “Medicine”, Rīga Stradiņš University, 16 Dzirciema Street, LV-1007 Rīga, Latvia; 2 Riga East Clinical University Hospital, 2 Hipokrata Street, LV-1038 Rīga, Latvia; laila.meija@rsu.lv; 3 Department of Public Health and Epidemiology, Rīga Stradiņš University, 9 Kronvalda bulvāris, LV-1010 Rīga, Latvia; olga.aizbalte@rsu.lv (O.A.); anna.piskurjova@rsu.lv (A.P.); 4 Faculty of Medicine, Rīga Stradiņš University, 16 Dzirciema Street, LV-1007 Rīga, Latvia; roberta.rezgale@rsu.lv (R.R.); vinita.cauce@rsu.lv (V.C.); 5 Institute of Food Safety, Animal Health and Environment “BIOR”, Lejupes Street 3, LV-1076 Rīga, Latvia; dzintars.zacs@bior.lv

**Keywords:** pregnancy, nutrition, fatty acids, n-3, n-6, erythrocytes

## Abstract

Background and Objectives: Dietary fats are essential for maternal and fetal health. Fatty acids (FAs) in erythrocytes characterize the FA profile, which is influenced by diet and other factors. The aim of this study was to evaluate the association between the main FAs in erythrocyte membrane phospholipids and their influencing factors—dietary fat and supplement intake and lifestyle factors—in Latvian pregnant women. Materials and Methods: This cross-sectional study included 236 pregnant and postpartum women. The data were collected from medical documentation, a food frequency questionnaire, and a questionnaire on demographic, lifestyle, health status, and nutritional habits in outpatient clinics and maternity departments. FAs in erythrocyte membrane phospholipids were determined using gas chromatography. Results: Correlations were found between dietary SFAs and erythrocyte SFAs (r = −0.140, *p* = 0.032) and PUFAs (r = 0.167, *p* = 0.01) and between dietary PUFAs and erythrocyte MUFAs (r = −0.143, *p* = 0.028). Dietary SFAs, MUFAs, and PUFAs positively correlated with the studied n-3 and n-6 FAs in erythrocytes. Vitamin D correlated positively with MUFA and negatively with total PUFA and AA in erythrocytes. There was a negative correlation between dietary vitamin A and linoleic acid in erythrocytes. Physical activity negatively correlated with erythrocyte MUFAs and positively with erythrocyte PUFAs. Alcohol consumption positively correlated with erythrocyte SFAs and negatively with erythrocyte PUFAs. Conclusions: There are indications that some dietary FAs may be correlated with erythrocyte FAs. Possible influencing factors for this association are alcohol, physical activity, vitamin D, and vitamin A.

## 1. Introduction

Lipids are one of the nutrients that play a key role in energy storage, tissue growth, and cell signaling, so the amount and quality of fat in a pregnant woman’s diet can affect fetal development and the long-term health of the mother and offspring [1].

Saturated fatty acids provide energy and mechanical protection, form energy reserves in our body, and participate in the absorption of fat-soluble vitamins. It is important to reduce the intake of such fatty acids during pregnancy to reduce the risk of cardiovascular disease, obesity, metabolic syndrome, and cancer. Adverse effects are associated with multiple mechanisms, including oxidative stress, endothelial dysfunction, promotion of insulin resistance and gestational hyperglycemia, elevation of blood pressure, development of thrombosis, and inflammation. Therefore, it is recommended to limit the amount of saturated fat in the diet, which is found in fatty dairy products, fatty meat, coconut, and palm oil [1,2,3].

The most common unsaturated fatty acids are n-3 and n-6 polyunsaturated fatty acids (PUFAs). Linoleic acid (LA) and α-linolenic acid (ALA) are considered essential dietary nutrients because they cannot be synthesized by mammalian cells, which lack the necessary enzymes to insert double bonds at specific positions. These fatty acids are precursors of a family of long-chain polyunsaturated fatty acids (LCPUFAs) with a 20 or 22-carbon chain. Through enzymes such as acyl-CoA synthetases, Δ6- and Δ5-desaturases, and elongases, LA is converted to arachidonic acid (AA), and ALA is converted to eicosapentanoic acid (EPA) and docosahexanoic acid (DHA) [4,5,6,7].

Both n-3 and n-6 LCPUFAs are involved in metabolic processes and the regulation of gene expression. They are essential for the development, angiogenesis, and vascularization of the placenta [8]. The embryo implantation process and the course of pregnancy and childbirth may be influenced by the eicosanoids (prostaglandins, leukotriens, and tromboxanes) derived from fatty acids. In general, the metabolites of n-3 LCPUFAs have mainly anti-inflammatory properties, whereas eicosanoids derived from n-6 LCPUFAs have mainly pro-inflammatory properties and may affect the development of complications, such as gestational diabetes and preeclampsia, during pregnancy [4,5,8]. In addition to showing anti-inflammatory properties, n-3 LCPUFAs also have antioxidant potential and can reduce oxidative stress [9]. These fatty acids (FAs) are necessary for the development of the fetal brain in the third trimester intrauterine and during the first year after birth and are found in the brain in large quantities [10]. The n-3 LCPUFAs also play a role in the development of the retina during pregnancy and psychomotor neurons in the first months of life [11].

Exposure to dietary FAs can be assessed via the determination of FAs in biological samples—adipose tissue, blood plasma, erythrocytes, and platelets [12]. Although adipose tissue is the best representation of long-term dietary FA intake, detection involves fat biopsy, which limits the widespread use of this method in epidemiological studies [13]. Blood plasma phospholipids serve as FA transporters and are not incorporated into cell membranes and thus do not necessarily reflect the FA composition of target tissues [13,14]. Considering the life span of erythrocytes (about 120 days), it has been concluded that the determination of FAs in erythrocyte membranes is a more objective measure than FAs measured in plasma, which only reflects the intake of FAs in a short period of time before the analysis is taken [12,13]. FA indices in erythrocyte membrane phospholipids are also suitable biomarkers to study correlations between fatty acid metabolism and disease [14].

Physiological and hormonal changes occur during pregnancy, which affects lipid metabolism and, therefore, the composition of FAs in target tissues [15,16]. The detection of FA in erythrocyte membrane phospholipids, as a potential biomarker, can reflect the risk of complications during pregnancy [17,18]. The FA composition of erythrocyte membrane phospholipids depends on the dietary intake of various FAs as well as age, ethnicity, genetics and endogenous metabolism, body mass index, health status including liver metabolism, lifestyle factors—smoking, alcohol consumption, physical activity—among other factors [16,17,18,19,20,21,22,23]. Thus, the aim of this study is to assess fat and FA intake—quantity and quality—and evaluate the association between the main FAs in erythrocyte membrane phospholipids and some of the influencing factors of this association—dietary fat intake, supplement intake, and the most significant lifestyle factors—in Latvian pregnant women.

## 2. Materials and Methods

### 2.1. Study Population

The sample was formed by conducting surveys of participants in outpatient institutions in Riga and stationary maternity wards in Latvian regions. Each participant was assigned an identification number for participation after signing the informed consent form. A total of 236 healthy pregnant women from 19 to 45 years in the 27th–40th week of gestation and postpartum women up to the 7th day after delivery took part in the study. The study included data from July 2020 to August 2021, inclusive.

The exclusion criteria for the study participants were as follows: age less than 18 years, place of residence outside Latvia, presence of a multiple pregnancy, and history of diabetes, celiac disease, inflammatory bowel disease, short bowel syndrome, or gastrointestinal tract and eating disorders. The extant scientific literature indicates notable distinctions in energy intake and eating behaviors between women with health conditions and those without, which can also affect the absorption of nutrients and consequently influence the results of fatty acids in red blood cells [24,25].

### 2.2. Data Collection

The food frequency questionnaire about the participants’ diet during the last six months (frequency and quantity of 211 food products) was adapted from the questionnaire of the Scientific Institute of Food Safety, Animal Health, and Environment “BIOR” for obtaining nutritional data on the population of Latvia [26]. The questionnaire includes 199 products and beverages, which are grouped into 20 product groups, as well as 12 supplement positions. For evaluation of the portion size of the food products and beverages, the “Photo Atlas of Food Products and Food Portions” was used. The questioning was performed by trained interviewers. As the food frequency questionnaire includes data on the diet of pregnant women in the last six months and that the need for energy increases by 300 kcal in the third trimester of pregnancy, participants who consumed less than 800 or more than 3800 kcal per day were excluded.

The questionnaire on demographic, lifestyle, health status, and nutritional habits included respondents’ anthropometric data (weight, height); demographic data (age, nationality, marital status, education, occupation); use of nutritional supplements during pregnancy (type, duration of use, dose); and lifestyle information (smoking, alcohol consumption, physical activity).

Both questionnaires were available in Latvian and Russian languages. In addition, data from medical documentation (anthropometric data, blood tests, and the course of pregnancy) were collected.

### 2.3. Blood Samples

Blood sampling was performed by certified laboratory nurses using a disposable needle and disposable containers. Venous blood tests were taken after the dietary survey: 4 mL of blood was drawn into an EDTA tube. The tube with blood was carefully mixed by inversion 5–10 times. The tube was then centrifuged for no longer than 5 min. Erythrocytes were separated from plasma by centrifuging the tubes for 10 min at 1000–2000 revolutions per minute at 4 °C. The EDTA-plasma supernatant, formed after centrifugation, was immediately transferred to another tube using a Pasteur pipette. During processing, the samples were kept at a temperature of 2–8 °C. The erythrocytes were mixed and distributed into three other tubes of 1 mL using a pipette. Within 4 h, the tubes containing collected erythrocytes were then frozen at −20 °C or lower for storage and without adding liquid. The frozen samples (−20 °C) were delivered to BIOR for further fatty acid analysis in frozen tubes within (−80 °C) for a maximum of 5 days. Fatty acid detection was conducted using GC model—Agilent 6890 N with a FID detector, sample set processed in Agilent Chemstation, column—SP-2560, 100 m × 0.25 mm × 0.20 µm (Supelco, Bellefonte, PA, USA). Chromatography heating conditions were the following: oven temperature gradient: 90 °C (held for 10 min) → 120 °C at 10 °C/min → 200 °C at 1.2 °C/min → 220 °C at 0.8 °C/min (held for 25 min). The injector’s temperature was 260 °C with a split ratio of 50:1. The carrier gas was Helium (purity 4.6). The detector’s temperature was 250 °C. For analysis, the gas flow was the following: hydrogen—40 mL/min, air—450 mL/min, and helium—1.0 mL/min. The total number of analyzed fatty acids was 26, and their identification of chromatographic peaks according to respective retention times (RTs) were determined by analytical standards of reported FA (e.g., Supelco 37 Component FAME Mix (CRM47885)).

The quantification method of fatty acids was external calibration without internal standards. All fatty acid (FA) peaks were subjected to integration, and subsequently, the proportional content of each FA component was determined as the percentage of its peak area relative to the total peak areas of all FA components.

### 2.4. Measurement of FA Composition in Erythrocytes

Fatty acids were determined in the phospholipids of erythrocyte membranes at the accredited laboratory at the Scientific Institute of Food Safety, Animal Health, and Environment “BIOR” by the gas chromatography (GC-FID) method.

First, the tube with the sample was taken out of the freezer for thawing. It was then mixed, and 200 µL was pipetted into another tube. Then, 800 µL of distilled water was added to a 200 µL erythrocyte sample and centrifuged for 10 min at 3000 rpm. The pellet containing phospholipid membranes was rinsed with 800 µL of distilled water and centrifuged again to obtain erythrocyte membranes. Then, 400 µL of distilled water and 3 mL of a chloroform–methanol solution (1:1 ratio) were added and mixed. The chloroform layer was transferred to another tube, and the solvent was removed by evaporation. Phospholipids were simultaneously hydrolyzed and methylated with a mixture of 100 µL butylhydroxytoluene and 0.5 mL boron triphosphide/methyl alcohol mixture for 60 min at 100 °C in closed tubes in a heating block. After cooling, 800 µL of distilled water and 800 µL of hexane were added for fatty acid extraction. The supernatant (hexane) containing the fatty acids was transferred to a clean glass container, evaporated to dryness under nitrogen gas, and re-dissolved in 100 µL of hexane for GC-FID analysis, a gas chromatography method. The results were expressed as a percentage of individual fatty acids out of total fatty acids.

### 2.5. Ethics

Ethical approval was obtained from the Clinical Research Ethics Committee of Riga Stradiņš University (No. 6-1/02/62). The study participants were provided with written information about the aim and objectives of the study. After a written agreement, participants were assigned a code for further data processing. Data obtained during the survey were encrypted with the code number assigned to each participant.

### 2.6. Data Analysis

Dietary data from the dietary frequency questionnaire were processed at the Scientific Institute of Food Safety, Animal Health, and Environment “BIOR” with the program developed at the Institute, based on the Microsoft Dynamics Ax 2009 program. This program uses the Latvian Food Composition Database, which was created to analyze food consumption data from the “Food Consumption Study of the Latvian Population (2012–2013)”, and which continues to provide data for other Latvian population food consumption studies. The program consists of algorithms and software designed to automate the computation of daily nutrient intakes and/or food group consumption [27].

SPSS version 24.0 was used for statistical data processing. Descriptive statistical methods—mean and median, dispersion indicators, standard deviation, interquartile range (Q1–Q3), and minimum and maximum values—were used to characterize the data. The relationship between the two variables was determined by applying Spearman’s correlation test. The difference in fatty acid composition between pregnant and postpartum women was compared using an independent *t*-test or Mann–Whitney U test. The difference in erythrocyte n-3 and n-6 fatty acid values based on alcohol use was compared using the Kruskal–Wallis test. The relationship between the two variables was determined by applying Spearman’s correlation test. A *p*-value < 0.05 was considered statistically significant.

## 3. Results

Table 1 shows the main background characteristics of the sample (age, socioeconomic status, BMI, nutritional status). A total of 53 (22.5%) pregnant women and 183 (77.5%) postpartum women were included in the study. Regarding the nutritional status, 22.7% (*n* = 54) of respondents were overweight, and 9.9% (*n* = 23) were obese; therefore, 32.6% (*n* = 77) of respondents exhibited overnutrition before pregnancy. Most of the participants—156 (66%)—were married, and 165 (69.6%) had higher education.

The data on exercise, alcohol use, and smoking status are summarized in Table 2. A total of 8.1% (*n* = 19) of women confirmed smoking during pregnancy, and 27.5% (*n* = 65) of women responded that they had consumed alcohol during pregnancy. A total of 49.4% (*n* = 32) confirmed the consumption of alcohol in the first trimester, with 24.1% (*n* = 16) and 26.6% (*n* = 17) confirming the same in the second and third trimesters, respectively.

Table 3 shows the intake of energy, nutrients, and the most relevant fatty acids of the sample. In the diet of the respondents, fats accounted for 41.7% of the total energy, proteins were 18.1%, and carbohydrates were 39.4%. Saturated fat accounted for 36.8% of the total fat.

Fatty acids were ingested from different food groups. Table 4 shows the dietary intake during the period studied. Additionally, with dietary supplements (multivitamins and fish oil), only two LCPUFAs were ingested: EPA—0.1 (0–0.14) g per day; DHA—0.06 (0–0.09) g per day.

The status of common n-3 and n-6 LCPUFAs in maternal erythrocyte phospholipids is shown in Table 5.

Table 6 provides a summary of the differences in erythrocyte fatty acid composition between pregnant and postpartum women. Statistical analysis revealed a significant difference in the levels of MUFAs, ALA, and AA between the two groups (*p* < 0.05).

The associations between dietary FA intakes and fatty acid composition (FAC) in erythrocyte membrane phospholipids are shown in Table 7. Dietary SFAs had a weak negative correlation with erythrocyte SFAs and a weak positive correlation with erythrocyte PUFAs, but dietary PUFAs showed a statistically significant weak negative correlation with erythrocyte MUFAs. In erythrocytes, dietary SFAs, MUFAs, and PUFAs were found to be positively correlated with n-3 and n-6 FAs. Dietary amounts of EPA and DHA had a weak positive correlation with erythrocyte DHA.

With nutritional factors (Table 8), vitamin D from the diet and food supplements had a weak positive correlation with MUFAs and a weak negative correlation with total PUFAs and AA in erythrocytes. There was also a negative correlation between dietary vitamin A and LA in erythrocytes.

No significant correlations were found between FAC in erythrocytes in respondents and different BMI and smoking status (*p*-values > 0.05). Physical activity correlated with erythrocyte MUFAs (r = −0.130; *p* = 0.046) and erythrocyte PUFAs (r = 0.154; *p* = 0.018). No association of physical activity with individual n-3 and n-6 fatty acids in erythrocyte membrane phospholipids was found. The amount of alcohol consumption had a weak positive correlation with erythrocyte SFAs (r = 0.151, *p* = 0.022) but a weak negative correlation with erythrocyte PUFAs (r = −0.151, *p* = 0.022). There was no statistically significant difference in erythrocyte n-3 and n-6 FA values depending on the status of alcohol consumption during pregnancy (*p* > 0.05).

## 4. Discussion

Inadequate macronutrients, including fat intake during pregnancy, are one of the nutritional factors that can negatively affect the health of both the mother and the fetus. Although their amount of energy intake was within the recommended limits, respondents did not consume enough carbohydrates, accounting for only 39% (recommended amount is 45–60%), whereas their fat intake (42%) exceeded the recommended amount (25–30%). In addition, pregnant women exceed the recommended amount of saturated fatty acids (up to 10% of the total amount of energy).

Our results were in line with the results of a Norwegian cohort study, in which a similar trend was found—insufficient intake of carbohydrates but excessive consumption of saturated fatty acids [28]. A committee of the Food and Agriculture Organization of the United Nations and the World Health Organization (WHO) recommends that PUFA intake should be 20 to 35% of fat during pregnancy. In turn, the daily intake of AA should be about 800 mg [29]. According to the results of our study, PUFAs accounted for 16.2% of total fat, and dietary AA was four times less than recommended. Although there is no clear consensus on the dietary requirements of LA and ALA during pregnancy, according to the Food and Nutrition Board of the Institute of Medicine, the daily intake should be 13 and 1.4 g per day, respectively; this was achieved by the study participants [30].

Few studies publish data on the amount of dietary fatty acids in the diet of pregnant women. Compared to [31,32,33], our participants consume more or similar amounts of LA, AA, ALA, EPA, and DHA.

The main sources of SFAs were milk, meat, and prepared foods, while the main sources of MUFAs and PUFAs were sauces. The results are difficult to compare because of the differing food groups and product databases used in the studies; although, when analyzing the sources of various FAs among the European population from 24 countries, the data show that the main sources of SFAs are not only meat and milk products but also fats, while PUFAs are mainly consumed with fats, meat and its products, and grains and cereal products [34,35].

The majority of erythrocyte membrane phospholipid FAs are SFAs, but MUFAs and PUFAs are found in smaller amounts, which is similar to the data of a Norwegian cross-sectional cohort study in which pregnant women (n = 247) participated in various gestational periods and from different geographical regions of Norway [36]. In a prospective hospital-based cohort study that investigated the fatty acid composition of erythrocyte membranes in 178 healthy, pregnant Japanese women in their third trimester, it was found that values of ALA, DHA, LA, and AA were higher, but EPA concentrations were lower compared to our study [37].

During our study, we observed that there are indications that dietary SFAs, MUFAs, and PUFAs can affect erythrocyte EPA and DHA levels and that these correlations were positive. Positive correlations were found between dietary SFAs and erythrocyte PUFAs as well as between dietary PUFAs and erythrocyte ALA, dietary MUFAs and erythrocyte LA, dietary PUFAs and erythrocyte LA, dietary SFAs and erythrocyte ALA, dietary MUFAs and erythrocyte ALA, dietary SFAs and erythrocyte LA, and of dietary SFAs, MUFAs, and PUFAs with erythrocyte AA. On the other hand, dietary SFAs had a weak negative correlation with erythrocyte SFAs and MUFAs. These data suggest that the consumption of some of these fatty acids affects FAC in erythrocyte membranes. The disparity in fatty acid profiles observed between pregnant and postpartum women can be partially attributed to the modulating effects of hormonal alterations [38].

In general, current data from other studies show that there is a positive correlation between maternal n-3 PUFAs in the diet, especially EPA and DHA, and their relative concentrations in erythrocytes. The level of AA, in turn, is inversely related to the amounts of EPA and DHA. Currently, there are conflicting data on whether dietary saturated and monounsaturated fatty acids can influence erythrocyte parameters, as endogenous FA de novo biosynthesis, elongation, and desaturation are important [17]. A large Danish national birth cohort study (n = 345) found a moderate correlation between dietary LCPUFAs and EPA in erythrocytes (r = 0.37, *p* = 0.001) [33]. In a cross-sectional study that included 895 pregnant women in 35–37 weeks of gestation, the intake of FAs was compared with the variables of six PUFAs in erythrocyte membranes—total n-3, total n-6, EPA, DPA, DHA, and ALA. A moderately high correlation between the amount of dietary PUFAs obtained from the food frequency questionnaire and the level of erythrocyte fatty acids was found only in EPA (r = 0.55) and DHA (r = 0.61) [39]. There are also data from Japanese pregnant women with correlation coefficients of r = 0.34 for EPA and 0.16 for DHA, in addition to a similar report comparing erythrocyte phospholipid fatty acid levels with dietary fatty acids in Mexican pregnant women, where higher coefficients were found for EPA and DHA (r = 0.36 and 0.35, respectively). These study data indicate that erythrocyte membrane phospholipid FAs are effective biomarkers to assess the intake of EPA and DHA; however, the association of individual dietary and erythrocyte FAs has been more thoroughly investigated [40].

Considering the endogenous metabolism of FAs, some of these FAs could be synthesized from other compounds in the body. In the current study, the degree of fat accumulation in the liver and liver enzymes was not characterized. Fat accumulation in the liver could affect FA levels in erythrocyte membranes, as it stimulates de novo SFA formation [20,41].

Body mass index is also one of the factors that could affect these indicators. Among respondents, one-third were overweight or obese before pregnancy, which is consistent with reports from other studies from Italy and the United States [42,43]. Although the literature often reports that BMI may influence erythrocytes n-3 and n-6, we did not find any association between dietary and erythrocyte FAs and BMI [42].

Vitamin D is known to affect lipid metabolism by lowering total cholesterol, triacylglycerols, and LDL cholesterol and increasing HDL cholesterol, but evidence is lacking to interpret the study data for erythrocyte fatty acid producers [44]. A higher amount of vitamin D taken from food and preparations was found to be associated with higher amounts of MUFAs but lower amounts of PUFAs and AA in erythrocyte membrane phospholipids.

No association was found between dietary fiber and erythrocyte fatty acids, which could be explained by the fact that the amount of fiber consumed was not very high. A high amount of dietary fiber can not only reduce fat absorption but also increase the amount of odd-chain fatty acids (15:0 and 17:0) in erythrocytes [45,46].

The fatty acid profile in erythrocytes is not related to the amount of fructose in the diet. However, it is known that in humans, 70% of fructose is metabolized in the liver, and a fructose-rich diet prompts de novo synthesis of fatty acids in the liver and triacylglycerols accumulation, thus serving as an important factor influencing liver functions. The possible explanation for the lack of association could be the relatively low consumption of fructose among the respondents; compared to the typical diet of the Western hemisphere, which is rich in sugars and contains an average of 49 g of fructose per day, the participants in our study consumed half as much [47,48]. Alcohol consumption during pregnancy is negatively associated with PUFA levels but positively associated with SFA levels in erythrocyte membrane phospholipids [17]. There is evidence that smoking may decrease the content of DHA and EPA in maternal erythrocytes, and in women who consume alcohol daily, higher alcohol intake was linked to lower concentrations of DHA and arachidonic acid AA in the plasma [49,50].

Physical activity can affect endogenous FA metabolism and, thus, erythrocyte MUFAs and PUFAs [17], and our study also demonstrated a weak correlation.

Currently, there have been few studies examining the correlations of groups of FAs, and the results of these studies have been inconclusive. The relationships between nutrition and erythrocytes n-3 and n-6 are the most studied because they are extremely important for fetal growth and development. Currently, the studies on dietary MUFA and SFA intake and its correlation with erythrocyte indices are limited in number and, moreover, have been conducted on other types of populations and cannot thus be directly applied to pregnant women. Data obtained from studies using serum fatty acids as biological samples cannot be compared with erythrocyte indicators from other studies.

A limitation of the study is that we analyzed only some of the factors influencing the FA profile. Fatty acids have differences in terms of absorption, tissue assimilation, and metabolism processes. These processes are influenced by many factors, such as genetic and endogenous fatty acid metabolism, smoking, alcohol consumption, and body mass index, as well as specific health conditions such as endocrine diseases and cardiovascular diseases, and other factors that can interact with each other to affect the concentration of fatty acids. Also, the used questionnaires have not been validated, which may have resulted in a measurement error.

In our study, we only assessed the association between body mass index and certain groups of fatty acids and specific fatty acids. However, we did not analyze the effect of weight gain during pregnancy. Excessive gestational weight gain is associated with various negative outcomes for both the pregnancy and the offspring. Available data from the Generation R study suggest that excessive gestational weight can impact plasmatic concentrations of SFAs, MUFAs, and n-3 PUFAs in blood plasma, but there are conflicting data about fatty acid levels in erythrocyte membrane phospholipids, which would reflect a longer intake period [49]. The population study of 1000 mother–infant pairs suggests that inadequate gestational weight gain is associated with increased DPA in erythrocytes but excessive gestational weight gain—with the decreased concentration of DHA [51].

The primary strength of this study lies in the characterization of FAs present in the phospholipids of erythrocyte membranes using a strategy widely recognized as the most accurate for evaluating the uptake of FAs in specific target tissues. It should be noted that there has previously only been a limited number of such studies. However, further studies are needed to evaluate all factors such as ethnicity, genetics, health status, liver function, smoking status, and alcohol consumption that may possibly affect the determination of fatty acid values in erythrocytes.

In the future, considering the established correlations and conducting further investigations to understand potential pathologies associated with alterations in fatty acid indicators, these indicators may serve as valuable biomarkers for various pregnancy complications. This could facilitate their timely recognition, enabling the initiation of appropriate therapeutic interventions encompassing dietary and lifestyle modifications.

## 5. Conclusions

Our results show that pregnant women consume an excessive amount of total and saturated fat. The results also suggest that dietary FAs play an important but often very complex role, not always direct, in affecting the erythrocyte FA status in pregnant women. Other possible influencing factors are alcohol, physical activity, vitamin D, and vitamin A but not BMI or smoking status.

The detailed mechanism behind the association of erythrocyte FAs with their influencing factors in pregnant women needs further clarification in the future, which could lead to the development of applications involving the use of erythrocyte FAs as biomarkers for prognostic and diagnostic purposes.

## Figures and Tables

**Table 1:** Participants’ baseline characteristics (*n* = 236).

Variable	Mean ± SD/*n* (%)
Participants	
Pregnant women	53 (22.5)
7-day post-postpartum women	183 (77.5)
Age, years	31.5 ± 5.0
Nationality	
Latvian	171 (72.5)
Russian	49 (20.8)
Other	16 (6.8)
Marital status	
Married	156 (66.0)
Live in partnership	78 (33.2)
Divorced	1 (0.4)
Widowed	1 (0.4)
Education	
Primary education/incomplete secondary education	13 (5.6)
General special/secondary/vocational education	41 (17.5)
Higher education	165 (69.6)
Incomplete higher education	15 (6.4)
BMI group	
Underweight	6 (2.6)
Normal weight	153 (64.8)
Overweight	54 (22.7)
Obese	23 (9.9)

BMI—body mass index, kg/m^2^.

**Table 2:** Characteristics of physical activity, alcohol use, and smoking during pregnancy (*n* = 236).

Variable	*n* (%)
Time spent walking and cycling per day during pregnancy	
<15 min	10 (4.3)
15–30 min	36 (15.4)
30–60 min	97 (41.5)
>60 min	91 (38.9)
Frequency of at least 30 min of physical exercise during pregnancy	
Daily	12 (5.2)
4–6 times a week	11 (4.7)
2–3 times a week	57(24.6)
Once a week	25 (10.8)
2–3 times a month	17 (7.3)
Some occurrences annually or less frequently, or never	106 (45.7)
I cannot exercise due to illness or disability	4 (1.7)
Physical activity at work	
I do not work	20 (8.5)
Very light	94 (40.0)
Easy	69 (29.4)
Medium	51 (21.7)
Heavy handwork	1 (0.4)
Alcohol consumption during pregnancy	
Yes	31 (13.5)
No	164 (71.6)
I had not consumed it after I found out about the current pregnancy	34 (14.8)
Smoking during pregnancy	
Yes	6 (2.5)
No	156 (66.1)
Yes, but quit at one week of pregnancy	13 (5.6)

**Table 3:** Daily intake of energy, nutrients, and fatty acids from foods and supplements.

Energy/Nutrient/Fatty Acids	Median (Q1–Q3)
Energy (kcal)	2286 (1730–2807)
Protein (g)	104 (81–129)
Carbohydrate (g)	225 (162–273)
Fiber (g)	23.6 (15.7–29.5)
Fat (g)	106 (78–132)
SFAs (g)	39.1 (27.8–50.0)
MUFAs (g)	38.9 (28.8–47.7)
PUFAs (g)	17.2 (11.6–21.6)
Vitamin A (µg)	1787 (1058–2156)
Vitamin D (µg)	45 (11–69)
Vitamin K (µg)	362 (260–449)
Vitamin E (mg)	13 (9–16)
n-3 FAs	
ALA (g)	2.5 (1.6–3.0)
EPA (g)	0.2 (0.1–0.3)
DHA (g)	0.3 (0.1–0.4)
n-6 FAs	
LA (g)	13.9 (9.1–12.6)
AA (g)	0.2 (0.2–0.3)

SFAs—saturated fatty acids; MUFAs—monounsaturated fatty acids; PUFAs—polyunsaturated fatty acids; FAs—fatty acids; ALA—α-linolenic acid; EPA—eicosapentanoic acid; DHA—docosahexanoic acid; LA—linoleic acid; AA—arachidonic acid.

**Table 4:** Daily median intake (Q1–Q3) of fatty acids (g) according to food groups.

Food Groups	SFAs, g	MUFAs, g	PUFAs, g	n-3, g			n-6, g	
				ALA, g	EPA, g	DHA, g	AA, g	LA, g
Jam	2.66 (0.74–3.14)	1.69 (0.62–2.36)	0.51 (0.08–0.64)	0.17 (0.02–0.10)	n/a	n/a	n/a	0.44 (0.05–0.51)
Milk and milk products	12.18 (6.71–16.10)	6.13 (3.48–8.04)	0.82 (0.48–1.03)	0.27 (0.16–0.36)	n/a	n/a	n/a	0.55 (0.32–0.68)
Fats	2.24 (1.14–2.63)	7.05 (3.86–8.99)	3.73 (1.12–5.09)	0.48 (0.09–0.57)	n/a	n/a	n/a	3.27 (1.02–4.52)
Meat and meat products	4.43 (2.43–5.56)	2.40 (1.07–3.25)	1.93 (1.04–2.48)	0.17 (0.09–0.2)	0.01 (0.01–0.02)	0.01 (0.01–0.02)	0.11 (0.06–0.14)	1.57 (0.86–2.05)
Cooked meat dishes	2.80 (1.12–3.80)	2.40 (1.08–3.25)	0.46 (0.20–0.60)	0.09 (0.04–0.13)	n/a	n/a	0.01 (0–0.01)	0.36 (0.14–0.47)
Fish	0.83 (0.18–1.26)	0.78 (0.17–1.14)	0.54 (0.10–0.74)	0.03 (0.01–0.04)	0.11 (0.01–0.16)	0.18 (0.02–0.25)	0.02 (0–0.02)	0.14 (0.02–0.19)
Eggs	1.18 (0.44–1.47)	1.54 (0.53–1.96)	0.58 (0.22–0.72)	0.10 (0.04–0.13)	n/a	0.05 (0.02–0.06)	0.4 (0.15–0.49)	0.04 (0.01–0.05)
Potatoes	1.01 (0.30–1.35)	0.88 (0.22–1.12)	1.23 (0.27–1.81)	0.26 (0.05–0.29)	n/a	n/a	0.01 (0–0.01)	1.03 (0.21–1.49)
Pancakes	0.64 (0.15–0.87)	0.75 (0.15–1.12)	0.17 (0.05–0.23)	0.03 (0.01–0.03)	n/a	n/a	n/a	0.14 (0.04–0.2)
Nuts and seeds	0.77 (0.09–0.85)	3.67 (0.41–3.68)	2.67 (0.27–3.27)	0.39 (0.02–0.48)	n/a	n/a	n/a	2.28 (0.22–2.64)
Sweets	6.49 (2.45–7.97)	3.38 (1.21–4.59)	1.38 (0.48–1.97)	0.23 (0.06–0.31)	n/a	n/a	0.01 (0–0.01)	1.13 (0.41–1.63)

SFAs—saturated fatty acids; MUFAs—monounsaturated fatty acids; PUFAs—polyunsaturated fatty acids; ALA—α-linolenic acid; EPA—eicosapentanoic acid; DHA—docosahexanoic acid; LA—linoleic acid; AA—arachidonic acid; n/a—not available.

**Table 5:** Fatty acid composition (% from total fatty acids) in erythrocyte phospholipids.

Fatty Acids	Median (Q1–Q3)
SFAs	49.7 (46.1–52.8)
MUFAs	18.6 (17.6–19.7)
PUFAs	28.5 (24.7–32.8)
n-3	
ALA	0.5 (0.4–0.7)
EPA	2.0 (1.7–2.5)
DHA	3.4 (2.7–4.1)
n-6	
LA	9.3 (7.8–10.8)
AA	6.7 (5.4–8.3)

SFAs—saturated fatty acids; MUFAs—monounsaturated fatty acids; PUFAs—polyunsaturated fatty acids; ALA—α-linolenic acid; EPA—eicosapentanoic acid; DHA—docosahexanoic acid; LA—linoleic acid; AA—arachidonic acid.

**Table 6:** Differences in fatty acid composition (% from total fatty acids) in erythrocyte phospholipids in pregnant and postpartum women.

Fatty Acids	Pregnant Women (*n* = 53)	Postpartum Women (*n* = 183)	*p*
	Median (Q1–Q3)	Median (Q1–Q3)	
SFAs	48.7 (46.1–51.8)	47.6 (46.0–53.0)	0.548 ^a^
MUFAs	19.4 (17.7–21.0)	18.3 (17.5–19.5)	0.002 ^a^
PUFAs	28.2 (26.1–32.0)	30.3 (24.0–32.9)	0.325 ^a^
n-3			
ALA	0.4 (0.3–0.6)	0.5 (0.4–0.7)	0.005 ^a^
EPA	2.1 (1.7–2.6)	2.0 (1.7–2.4)	0.106 ^b^
DHA	3.6 (2.5–4.4)	3.4 (2.7–4)	0.580 ^b^
n-6			
LA	9.5 (7.9–11.3)	9.5 (7.7–10.6)	0.537 ^a^
AA	6.7 (5.5–7.7)	7.4 (5.2–8.5)	0.046 ^a^

^a^ Independent Mann–Whitney U test was used to compare means between groups. ^b^ Independent *t*-test was used to compare means between groups. SFAs—saturated fatty acids; MUFAs—monounsaturated fatty acids; PUFAs—polyunsaturated fatty acids; ALA—α-linolenic acid; EPA—eicosapentanoic acid; DHA—docosahexanoic acid; LA—linoleic acid; AA—arachidonic acid.

**Table 7:** Correlation between dietary fatty acid intakes and fatty acid concentration in erythrocyte membrane phospholipids ^a^.

Dietary Fatty Acids, g	FAs in Erythrocytes, %							
	SFAs	MUFAs	PUFAs	n-3			n-6	
				ALA	EPA	DHA	LA	AA
SFAs	−0.140 *	0.006	0.167 *	0.588 **	0.156 *	0.207 **	0.619 *	0.548 **
MUFAs	−0.103	−0.056	0.110	0.686 **	0.245 **	0.312 **	0.758 **	0.559 **
PUFAs	−0.030	−0.143 *	0.084	0.776 **	0.211 **	0.290 **	0.984 **	0.495 **
n-3								
ALA	−0.046	−0.085	−0.066	−0.067	0.001	0.074	−0.010	−0.018
EPA	0.021	−0.029	−0.034	−0.010	0.074	0.174 *	−0.069	−0.181
DHA	−0.020	0.057	0.021	−0.026	0.077	0.157 *	−0.068	−0.087
n-6								
LA	−0.145	−0.109	−0.083	−0.086	−0.054	0.025	0.029	0.043
AA	0.093	0.033	0.02	−0.041	0.012	0.063	−0.044	0.038

^a^ All values are presented as Spearman rank correlation coefficients; * *p* < 0.05; ** *p* < 0.01. SFAs—saturated fatty acids; MUFAs—monounsaturated fatty acids; PUFAs—polyunsaturated fatty acids; ALA—α-linolenic acid; EPA—eicosapentanoic acid; DHA—docosahexanoic acid; LA—linoleic acid; AA—arachidonic acid.

**Table 8:** Correlation between nutritional factors and fatty acid concentration in erythrocyte membrane phospholipids ^a^.

Dietary Factors	FAs in Erythrocytes							
	SFAs	MUFAs	PUFAs	n-3			n-6	
				ALA	EPA	DHA	LA	AA
Fiber, g	0.101	−0.082	−0.072	0.008	−0.039	0.005	−0.081	−0.137
Fructose, g	0.073	−0.050	−0.072	0.023	−0.127	−0.076	−0.024	−0.070
Vitamin A, µg	−0.44	−0.55	0.78	−0.074	−0.025	0.110	−0.145 *	−0.003
Vitamin D, mkg	0.091	0.133 *	−0.168 *	−0.034	0.091	0.123	0.124	−0.149 *
Vitamin E, mg	0.009	−0.106	0.041	−0.086	−0.038	0.042	−0.108	−0.037
Vitamin K, µg	0.019	−0.38	−0.24	−0.03	−0.04	0.022	−0.092	−0.098

^a^ All values are presented as Spearman rank correlation coefficients; * *p* < 0.05. SFAs—saturated fatty acids; MUFAs—monounsaturated fatty acids; PUFAs—polyunsaturated fatty acids; ALA—α-linolenic acid; EPA—eicosapentanoic acid; DHA—docosahexanoic acid; LA—linoleic acid; AA—arachidonic acid.

## Data Availability

The data presented in this study are available upon request from the corresponding author.
